# The hows and whys of face memory: level of construal influences the recognition of human faces

**DOI:** 10.3389/fpsyg.2015.01524

**Published:** 2015-10-07

**Authors:** Natalie A. Wyer, Timothy J. Hollins, Sabine Pahl, Jean Roper

**Affiliations:** School of Psychology, University of PlymouthPlymouth, UK

**Keywords:** construal level, face recognition, configural processing, face inversion effect

## Abstract

Three experiments investigated the influence of level of construal (i.e., the interpretation of actions in terms of their meaning or their details) on different stages of face memory. We employed a standard multiple-face recognition paradigm, with half of the faces inverted at test. Construal level was manipulated prior to recognition (Experiment 1), during study (Experiment 2) or both (Experiment 3). The results support a general advantage for high-level construal over low-level construal at both study and at test, and suggest that matching processing style between study and recognition has no advantage. These experiments provide additional evidence in support of a link between semantic processing (i.e., construal) and visual (i.e., face) processing. We conclude with a discussion of implications for current theories relating to both construal and face processing.

## Introduction

It is well-established that the ability to recognize human faces is optimized under conditions that allow people to make use of configural information. That is, accurately identifying another person relies not simply on recognizing individual features (their nose, their eyes, etc.) but on recognizing the way that those features are spatially arranged to make the complete face. Factors that disrupt or distort that configural information—or make it more difficult for people to take advantage of it—invariably impair face recognition for most people (Yin, 1969; Young et al., 1987).

For example, prior research has demonstrated that participants who had viewed a video portraying a crime were more accurate at identifying the perpetrator if they had first identified the “global” shape of so-called Navon letters (i.e., large letter shapes composed of smaller constituent letters) than if they had first identified the “local” features of Navon letters (Macrae and Lewis, 2002; Perfect, 2003; Perfect et al., 2008). Likewise, Martin and Macrae (2010) found that individual differences in “global precedence” (i.e., the extent to which people find it easier to identify the global shape than the local features of Navon letters) also predicted face recognition performance. Hence, there appears to be a reliable link between processing visual-spatial stimuli such as Navon letters and processing human faces. To the extent that people process other visual stimuli in a more configural fashion, the better they are at taking advantage of configural information in faces.

In contrast, experimental manipulations that shift attention away from configural information and toward facial features have been shown to disrupt face recognition. For example, work on verbal overshadowing (Schooler and Engstler-Schooler, 1990)—the phenomenon wherein providing a verbal description of a face interferes with its later recognition—suggests that the tendency to describe faces in terms of their features promotes a shift in processing (from configural to feature-based) which later impairs optimal face recognition (Schooler, 2002).

One assumption reflected in much of this work is that faces are, by default, processed in a configural manner by most people. Thus, when feature-based processing is interposed between exposure to a face and its later recognition, the mis-match between processing styles at study and at test is in part responsible for the disruption in recognition performance. In support of this view, Lewis et al. (2009; see also Weston et al., 2008) manipulated global vs. local processing independently (using the standard Navon manipulation) before a multiple-face study phase and again prior to a recognition test, and reported that recognition performance was superior when processing styles at study and test matched (e.g., when a global Navon manipulation at study was paired with a global (vs. local or control) Navon manipulation at test).

While configural processing may often be adopted when viewing a face, recent evidence suggests that use of configural information during face recognition may also be influenced by a general disposition to process information (by default) in a more global fashion. Wyer et al. (2012) reported that individual differences in autism-related traits [as measured by the Autism Quotient (AQ) scale; (Baron-Cohen et al., 2001)] predicted the magnitude of the face-inversion effect (FIE; Yin, 1969). The face inversion effect refers to the established finding that perceivers' ability to recognize faces that they have seen before is diminished when those faces are presented upside-down. This effect appears to be driven by the fact that the configural information that is present when people encounter a face in its natural upright position is disrupted when that face is inverted. Indeed, manipulations that diminish perceivers' reliance on configural information also diminish the face inversion effect (Farah et al., 1995). Wyer et al. (2012) posited, and found evidence to support, that individuals who possessed high levels of autism-related traits are less able to benefit from the availability of configural information when present in upright faces, and hence showed a smaller difference in their ability to recognize upright vs. inverted faces.

Wyer et al.'s findings that individual differences in autism-related traits predicted the magnitude of the face inversion effect provide evidence that differences in non-visuospatial processing might be associated with the way that people process faces. That is, unlike studies using Navon letters to measure or manipulate global processing on a visual-spatial task, the measures used in these studies (AQ and Empathizing and Systematizing Quotients [EQ-SQ]) do not measure respondents' preferences or abilities in the visual-spatial domain. While perhaps surprising, this finding is consistent with theories of autism spectrum disorders (ASD) that posit that individuals with ASD (and by extension, individuals who score highly on the AQ or EQ-SQ scales) have weak central coherence—that is, they lack “the tendency to draw together diverse information to construct higher-level meaning in context” (Frith and Happé, 1994, p. 121) that is shown by non-ASD individuals (and those with low AQ or EQ-SQ scores).

The possibility that face recognition deficits among ASD individuals derive from a more general inability to integrate information suggests that a common mechanism may underlie both visual and non-visual (e.g., conceptual or semantic) processing. If that is the case, then other measures or manipulations of global processing might also be expected to influence face processing in general and the emergence of a face inversion effect in particular. One such manipulation that has received considerable attention in the social cognition literature is level of construal. Construal Level theory posits that any event can be viewed—or construed—in more general/abstract or more specific/detailed ways. For example, driving a car might be construed as making progress toward your destination (high-level construal) or as using the steering wheel, accelerator, and clutch (low-level construal). Research stemming from Construal Level theory has established that manipulations that induce high-level or low-level construal impact a wide range of social and cognitive tasks. For example, Smith and Trope (2006) reported that the concept of power (a form of social distance associated with high-level construal) altered performance on visual tasks.

Construal level has also been recently linked to social memory, including memory for faces. Wyer et al. (2010) obtained evidence that manipulations of temporal distance influenced performance on a face recognition task. Participants who were asked to think about an event in the near future (requiring low-level construal; Trope and Liberman, 2003) were less accurate in identifying a confederate they had encountered earlier in the experiment than were those who were asked to think about the same event in the distant future (encouraging high-level construal). The authors also reported that face recognition accuracy was correlated with more direct measures of construal (i.e., the extent to which participants reported thinking about future events in abstract vs. detailed terms (Study 1) and the extent to which they categorized items in a more vs. less inclusive manner (Study 2). This is consistent with the possibility that high-level construal induces global processing and by extension facilitates configural processing, which benefited face recognition (see also Hunt and Carroll, 2008, for evidence that imagining a temporally distant event overrides the verbal overshadowing effect).

Yet, correlations of construal with face recognition accuracy are only suggestive of a link between construal level and configural processing. More compelling would be evidence that construal level had a differential effect on face recognition under conditions where configural information was either intact or disrupted. The present study was designed to provide just such a test.

## The present research

Here, we report the results of three experiments in which construal level is manipulated prior to recognition (Experiment 1), during study (Experiment 2) or independently at both study and recognition (Experiment 3). In each study, the recognition test included both upright and inverted faces, allowing us to assess the extent to which participants made use of configural information in order to evaluate whether a face had been previously encountered or not.

We note that all experiments reported here were approved by the Ethics Committee of the Faculty of Science and Technology at the University of Plymouth. Written informed consent was obtained from all participants prior to beginning the study, which complied with recommendations of the American Psychological Association for the ethical treatment of human participants.

## Experiment 1

In our first experiment, participants viewed a set of faces during a study phase without manipulation of construal. Prior to completing a recognition task, they engaged in a task designed to induce low-level or high-level construal. In the test phase, they were asked to identify which of a set of old and new faces they had seen before. Critically, half of the faces were presented inverted at test.

Our expectation was that an induction of high level construal would produce greater inversion effects in face recognition. This rests upon two assumptions that derive from the literature discussed above. The first is that construal level will influence how people process facial stimuli: high level construal (“why” questions) will encourage configural processing whilst low-level construal (“how” questions) will encourage featural processing. The second assumption is that performance will be enhanced to the extent that the processing orientation adopted matches the information afforded by the test stimulus. For upright faces, a bias toward configural processing will tend to enhance performance, because people can easily access the configural information within the image. However, bias toward configural processing will have a much smaller effect on inverted faces because there is less useful configural information to access. Bias toward featural processing on the other hand is likely to impair performance on upright faces because it leads to the relative neglect of useful configural information, whilst having little impact upon performance on inverted faces. Thus, relative to control, we anticipate that a configural processing bias resulting from high-level construal should cause a larger inversion effect, whilst featural processing bias resulting from low-level construal should cause a smaller inversion effect. In both cases, consistent with previous research (e.g., Wyer et al., 2010), the pattern should largely be driven by changes in performance on upright faces, rather than inverted faces.

### Methods

#### Participants and design

Participants were 74 undergraduate students^1^ (51 female, *M*_*age*_ = 28 years, *SD* = 12.68) who took part in the study in partial fulfillment of a course requirement or were paid £4 (approximately $6) for their participation.

Sample size was determined using GPower with an estimated effect size (f) of 0.63 (derived from Lewis et al., 2009, Experiment 1, control condition) which indicated a required total sample size of 45 (*n* = 15 per between-participants condition) to achieve power of 0.95. Because the construal manipulation used in this study was arguably a more subtle manipulation than the Navon task used in Lewis et al. (2009), we increased the sample by approximately 50% (*n* = 24 per between-participants condition). Participants were randomly assigned to one of three conditions: high-level construal, low-level construal, or control.

#### Procedure

Participants were tested in groups of up to three people. Each participant sat in an individual cubicle containing a computer. They entered demographic information and then the computer programme began. Participants were shown a sequential presentation of 20 Caucasian faces from Minear and Park's (2004) face database (equal numbers of male/female, age range 20–30) for 3 s per image, with an inter-stimulus interval of 500 ms. The images showed head and shoulders only and each person was wearing a black tee shirt.

##### Construal manipulation

After viewing the faces, participants were directed to a workbook containing a pen and paper task (adapted from Freitas et al., 2004) where they were asked to fill in a series of three boxes to describe either “why” (high-level construal) or “how” (low-level construal) they would (a) “maintain physical health,” (b) maintain stable finances, and (c) “maintain personal relationships.” In the high-level condition the instructions were as follows: “This exercise is intended to focus your attention on *why* you do the things you do. You will be asked to fill in a series of boxes and for each subsequent box to generate a ‘why’ answer. For example if we had given you the subject ‘do well in school’ your first ‘why’ answer might be ‘to get a good job’. The next box up would be asking you to consider *why* you would get a good job. It might be that you think you would get a good job to ‘be successful’. In the box above that you would have to think about *why* you would want to be successful and this could be ‘to have a happy life’.” In the low-level condition the instructions were as follows: “This exercise is intended to focus your attention on *how* you do the things you do. You will be asked to fill in a series of boxes and for each subsequent box to generate a ‘how’ answer. For example if we had given you the subject ‘do well in school’ your first ‘how’ answer might be ‘study hard’. Your next box down would be asking you to consider *how* you would study hard. It might be that you think you could study hard by ‘going to the library’. In the box below that you would have to think about *how* you would go to the library and this could be by ‘walking down the street’.” Participants then completed a diagram containing three response boxes, each building on the previous response. The direction of boxes varied between condition (bottom up for the high-level condition and top down for the low-level condition).

Participants in the control condition spent an equivalent amount of time (4 min, which was determined by prior use of the construal manipulation in our laboratory) playing with play dough provided by the experimenter. This activity was chosen to fill the interval between study and test because it should not induce either high-level or low-level construal.

##### Test phase

Following the manipulation participants pressed the space bar to begin the test phase of the study. Participants were asked to view 40 faces presented sequentially and in random order. These included the 20 images seen during the study phase, along with 20 new images (10 male 10 female). For each face presented participants had to indicate whether they had seen the face before by pressing “y” for yes or “n” for no on the computer keyboard. Half of the original faces and half of the new faces were inverted (including equal numbers of male/female faces). The test phase was self-paced, with each successive image appearing once the participant had made their judgment. At the end of the experiment participants were thanked for their participation and given a full written debrief of the nature of the study.

### Results

**Figure 1:**
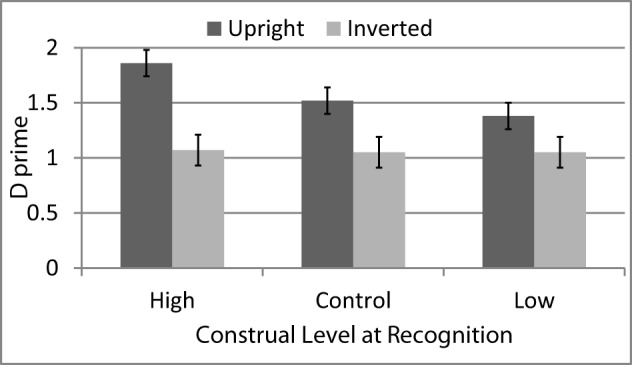
**Face recognition accuracy (average d′ with standard errors) for upright vs. inverted faces following a construal level manipulation (Experiment 1)**.

One participant's data was excluded as he spent over three times longer on the construal manipulation task than the average^2^. Recognition test performance was analyzed using independent indices of discrimination (d′) and response criterion (c)^3^. D′ scores were computed separately for upright and inverted faces for each participant. For d′ calculations in this and subsequent experiments, hit rates of 0 were replaced by 1/n (e.g., in this study, 1/10 or.10) and hit rates of 1 were replaced with n-1/n (e.g., in this case, 9/10 or.90). D′ scores for each face orientation were then entered as repeated measures in a mixed-model Analysis of Variance (ANOVA) where Construal (High vs. Low vs. Control) was entered as a between-participants factor and Face Orientation (Upright vs. Inverted) as a within-participants factor. Mean discrimination in each condition, for upright and inverted faces, is shown in Figure 1. The main effect of Construal was not significant, *F*_(1, 70)_ = 1.44, *p* = 0.24, η_*p*_^2^ = 0.04, *CI:* 0, 0.12. However, the analysis yielded a significant main effect of Face Orientation, *F*_(1, 70)_ = 41.02, *p* < 0.001, η_*p*_^2^, *CI:* 0.19, 0.51, which was qualified by a marginally significant interaction with Construal Level, *F*_(2, 70)_ = 2.78, *p* < 0.07, η_*p*_^2^ = 0.07, *CI:* 0, 0.19. Specifically, while participants in all conditions showed a face inversion effect, the magnitude of the effect was considerably greater in the high-level construal condition (*M*_*upright*_ = 1.86, *SE* = 0.12, *M*_*inverted*_ = 1.07, *SE* = 0.14) than in the low-level construal condition (*M*_*upright*_ = 1.38, *SE* = 0.12; *M*_*inverted*_ = 1.05, *SE* = 0.14) with the control condition falling in between (*M*_*upright*_ = 1.52, *SE* = 0.13 vs. *M*_*inverted*_ = 1.05, *SE* = 0.13).

Simple effects analyses confirmed that the effect of face inversion was strongest in the high-construal condition, [*F*_(1, 70)_ = 31.54, *p* < 0.001, η_*p*_^2^ = 0.31, *CI*: 0.14, 0.46] and the weakest in the low-level construal condition [*F*_(1, 70)_ = 5.31, *p* = 0.024, η_*p*_^2^ = 0.07, *CI:* 0, 0.20] with control in between [*F*_(1, 70)_ = 10.32, *p* = 0.002, η_*p*_^2^ = 0.13, *CI:* 0.02, 0.28]. Further, this difference appears to be driven by the effect of Construal on recognizing upright faces [*F*_(2, 70)_ = 3.86, *p* < 0.03, η_*p*_^2^ = 0.10, *CI:* 0, 0.23] as there were no differences among conditions when it came to recognition of inverted faces [*F*_(2, 70)_ = 0.01, *p* = 0.99, η_*p*_^2^ < 0.01]. For upright face recognition, paired comparisons among the three construal conditions indicated that high-level construal led to superior performance than both low-level construal (*p* < 0.01) and control (*p* = 0.06), which did not differ from each other (*p* = 0.46)^4^.

An additional exploratory analysis investigated whether the effect of Construal dissipated over the course of the test phase. A Three-Way mixed-model ANOVA was carried out on accuracy rates, in which Construal (high-level vs. low-level vs. control) was a between-participants factor and Face Orientation (upright vs. inverted) and test-half (1st half vs. 2nd half) were repeated measures. Test-half had no effect on overall performance [*F*_(1, 70)_ = 2.76, *p* = 0.10, η_*p*_^2^ = 0.04] and did not moderate the Construal level X Orientation interaction [Three-Way interaction *F*_(2, 70)_ = 0.08, *p* = 0.93, η_*p*_^2^ < 0.01] which remained significant, *F*_(2, 70)_ = 3.70, *p* = 0.03, η_*p*_^2^ = 0.10.

### Discussion

The results of this experiment support the hypothesis that construal level affects the way that faces are processed at test, and specifically that high-level construal promotes global processing, making people better at taking advantage of the configural information that is offered by a face. Low-level construal, on the other hand, diminishes that ability so that people are not much better at recognizing upright than inverted faces. Under control conditions, participants were able to benefit from the configural information that was available in upright faces. Inducing high-level vs. low-level construal appeared to increase or decrease this ability relative to control, perhaps by maximizing attention to configural information (in the former case) or shifting attention toward features instead (in the latter).

These findings are notable for a number of reasons. First, whilst previous experiments have imposed verbal manipulations that have impacted upon face processing (e.g., verbal overshadowing; Schooler and Engstler-Schooler, 1990), they have done so by requiring participants to verbally describe a visual stimulus. The present experiment is perhaps the first to demonstrate that an entirely non-visual-spatial manipulation of processing style that was completely unconnected in content to the stimuli influences performance on an old-new face recognition test. Moreover, these findings build on those reported by Wyer et al. (2010) by providing concrete evidence regarding the extent to which construal level affects face recognition due to its influence on configural processing. By employing a face inversion paradigm, we are able to provide a stronger link between construal level and the ability to use configural information. Finally, this study suggests that construal level—once imposed—has a fairly resilient effect on processing, as a single manipulation, lasting only a few minutes, was sufficient to influence recognition performance over a large number of trials.

One implication of these results is that differences in face recognition success may be based on the extent to which processing style, and construal level in particular, at study is the same as it is at test. If most of our participants employed configural processing by default during the study phase, we might assume that they were in something equivalent to a high-level construal condition—meaning that those who were assigned to the high-level condition during the retention interval would have experienced a match in processing styles whereas those assigned to the low-level condition would have experienced a mis-match (and consequently performed more poorly).

On the other hand, the aforementioned research by Wyer et al. (2012; see also Richler et al., 2011a; DeGutis et al., 2013) suggests that individuals may vary in their default mode of processing in response to seeing a face. For example, those low in AQ likely employ global processing (in line with high-level construal) whereas those high in AQ may be more likely to employ feature-based processing (in line with low-level construal). In that case, the construal manipulation during the retention interval may have interacted with participants' processing style at study such that the low-level construal manipulation had a particularly detrimental effect on those participants who would otherwise employ configural processing during the recognition test. Likewise, the high-level construal manipulation may have had a particularly beneficial effect on those participants who would otherwise engage in feature-based processing. This interpretation would suggest that it is not the match vs. mismatch in processing style that determines recognition success, but the extent to which participants are encouraged to employ configural processing.

## Experiment 2

In our second experiment, we sought further evidence regarding the influence of construal level on face processing and recognition. As noted above, one interpretation of the results obtained in Experiment 1 is that high-level construal improved face recognition because it encourages configural processing at test, whereas low-level construal undermines recognition performance because it shifts attention away from configural information at test. If this is the case, one might expect that altering the construal level that is in operation when a face is initially encountered might also influence its subsequent recognizability. That is, high-level construal may promote optimal processing of a face's configural information whilst low-level construal may result in relatively impoverished processing. Such differences might then be reflected in the overall likelihood that faces studied under different levels of construal are correctly recognized subsequently. Moreover, based upon the same argument as advanced for Experiment 1, one might expect the magnitude of the inversion effect to be altered by the orienting task prior to study because faces for which configural information is optimally processed should show a greater advantage for upright over inverted face recognition, with less of a difference for faces for which configural information was not processed at study.

Conceptually, the idea for this experiment replicates previous work involving Navon letters as an orienting task. Two studies have manipulated the orientation toward global or local Navon letters prior to study and test. Lewis et al. (2009) found a significant study-test interaction, such that recognition was highest when the processing orientation prior to study matched the orientation adopted prior to test. That is local study—local test and global study—global test both outperformed mismatching study and test. However, they found no main effect of orientation at study alone. A similar procedure was used by Weston et al. (2008) with a different outcome. They found effects of pre-test orientation, but no effect of orientation prior to study, and no interaction. Thus, contrary to a straightforward account, neither study strongly supported the idea that increased configural processing at study will lead to superior face recognition. However, neither study involved inverted faces at test, so there was no direct measure of the impact upon access to configural information.

There are studies which have examined the effects of processing style at study on subsequent recognition of upright and inverted face, but these have used direct manipulations rather than transfer effects from a prior orienting task. Valentine and Bruce (1986, Experiment 3) had participants judge study upright faces and houses either in terms of a distinctive feature, or in terms of a descriptive trait, designed to produce featural or configural processing respectively. Both kinds of items were subsequently tested upright or inverted. The faces, but not houses, showed an inversion effect, and the size of the inversion effect for faces was not moderated by the kind of processing at study. McKelvie (1996) tested face-recognition only, but participants judged faces at study either by feature or by trait prior to a test of upright or inverted face recognition in three experiments. The results suggested that inversion is sensitive to processing manipulations at study that encourage participants to process the face as a whole, as would be predicted, but that personality-based processing does not elicit this kind of encoding.

In summary, the prior research on the effects of manipulations of encoding is mixed. Collectively, the studies which have attempted to manipulate configural or featural encoding of faces directly have not resulted in the pattern anticipated, namely a larger inversion effect following configural encoding. The only study to demonstrate such a pattern was McKelvie (1996) Experiment 3, which manipulated the focus of attention to the face. The two studies to look for transfer effects from orientation toward Navon letters found no main effect of encoding, but neither involved inversion at test. Any prediction is therefore somewhat speculative. If the effects of construal at encoding mirror those seen at test in Experiment 1 then our expectation would be that Experiment 2 should replicate the pattern seen in Experiment 1. That is, inducing high level construal trial-by-trial during study would result in more configural processing of faces at encoding, which in turn would lead to superior memory for upright faces at test, with little impact of performance on inverted faces.

### Methods

#### Participants

Participants were 31 individuals (17 females, *Mage* = 30.4 years, *SD* = 12.82) from the Plymouth community who received £4 (approximately $6) for taking part in a 30-min experiment^5^. Sample size was estimated based on the results of Experiment 1. Prior research (e.g., Weston et al., 2008; Lewis et al., 2009) has not demonstrated main effects of manipulated processing style at encoding on later recognition performance; thus, a target effect size is difficult to determine. We judged that any effect would likely be smaller than that observed in Experiment 1, and increased the sample size (*n* = 31 for the entirely within-participants design) by approximately 1/3 in order to compensate for this. Participants were tested individually or in small groups.

#### Design and procedure

##### Study phase

Each participant sat in a cubicle containing a computer. Participants were told the study was looking at the effect of awareness of the presence of others on perceptions of ordinary everyday events. They were then shown a 24 faces each one presented for 3 s (equal numbers of male/female). Faces used were drawn from the same database as in Study 1 (Minear and Park, 2004). Preceding each face, participants were asked to type a sentence in answer to either a “how” question (e.g., “How do you care for a houseplant?”) or a “why” question (e.g., “Why do you clean the house?”). As a control condition some faces were preceded by a request to retype five words (e.g., “Please retype the words: chair, soup, tree, fish, roof”). These processing tasks were self-paced, and the subsequent face image appeared once the participant had pressed the return key. After presentation of all 24 faces, participants were instructed to wait silently for 2 min until the test phase began.

##### Test phase

In the test phase participants were asked to view 48 faces presented sequentially (the 24 original faces plus 24 new faces). In all other aspects, the test phase was identical to Experiment 1, as was the debriefing procedure.

### Results

**Figure 2:**
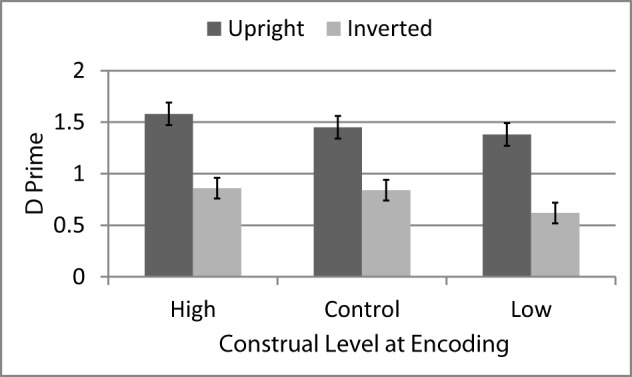
**Face recognition accuracy (average d′ with standard errors) for upright vs. inverted as a function of construal level at encoding (Experiment 2)**.

D′ scores were computed separately for upright and inverted faces that had been encoded in the high-level construal, low-level construal, or control condition during the study phase^6^, and are shown in Figure 2. D′ scores were entered into a Two-Way ANOVA with Face Orientation and Construal both entered as repeated measures. This analysis yielded a significant main effect of Face Orientation, *F*_(1, 30)_ = 39.54, *p* < 0.001, η_*p*_^2^ = 0.57, *CI:* 0.30, 0.71, such that participants were more accurate for upright faces (*M* = 1.47, *SE* = 0.09) than for inverted faces (*M* = 0.77, *SE* = 0.09). There was also a main effect of Construal, *F*_(2, 60)_ = 4.53, *p* < 0.02, η_*p*_^2^ = 0.13, *CI:* 0.01, 0.28, such that accuracy was highest for faces encoded in the high-level construal condition (*M* = 1.22, *SE* = 0.08) and lowest for those encoded in the low-level construal condition (*M* = 1.00, *SE* = 0.09) with faces encoded in the control condition falling in between (*M* = 1.14, *SE* = 0.08). The effect of Construal was not moderated by Face Orientation, *F*_(2, 60)_ = 0.53, *p* = 0.59, η_*p*_^2^ = 0.02, *CI:* 0, 0.10.

### Discussion

In Experiment 2, we found that construal level—this time manipulated prior to encoding—influenced participants' later ability to recognize *both* upright and inverted faces. This was not the pattern we anticipated on the basis of Experiment 1, but we noted that previous attempts to manipulate processing orientation at encoding had produced mixed findings with regards subsequent recognition of upright and inverted faces. Descriptively, the effect is quite straightforward. It appears that low-level construal results in weaker memory traces for the studied faces than control or high-level construal, and that these traces impact equally upon the ability to recognize upright or inverted faces. However, it is hard to attribute this pattern uniquely to changes in either configural or featural processing alone. If the effect were due to configural processing, we would have anticipated an interaction with inversion, as in Experiment 1. Conversely, if we take performance on inverted faces as an index of featural processing, then we would predict superior performance following low level encoding compared to high level encoding, which is the reverse of what was observed. Rather, low-level construal appeared to result in relatively impoverished encoding of *both* facial features and their configurations. Thus, whilst the construal manipulation influenced face recognition performance, it appeared to do so through a general mechanism, such as overall attention or motivation at study.

Although the results of Experiment 2 are consistent with there being an overall advantage of high-level construal when it comes to face processing, they do not conclusively rule out the matching hypothesis discussed in the introduction and in relation to the results of Experiment 1. The matching hypothesis suggests that when construal level at encoding matches construal level at test, recognition performance is maximized. In the present experiment, given the absence of any added manipulation during the interval between the study phase and the recognition test, it is possible that most participants reverted to their default mode of processing at test. If, for most participants, this entailed a shift (or return) to configural processing, this would have equated to adopting a high-level construal—resulting in a processing match when it came to recognizing faces that had been encoded in the high-level construal condition, and a mismatch when recognizing faces that had been encoded in the low-level construal condition.

## Experiment 3

In our third and final experiment, we wished to further differentiate between these two possibilities: (a) that face encoding and recognition are both enhanced by high-level construal (and undermined by low-level construal) vs. (b) that face recognition is optimized when construal level at recognition matches that at encoding. Thus, in this experiment, we independently manipulated construal level both at encoding and at recognition. If face recognition is best when construal level at study matches construal level at test, then we should actually see an advantage for participants who carry out the recognition test under low-level construal *when it comes to recognizing faces encoded under low-level construal conditions*. In contrast, if low-level construal interferes with all aspects of face processing, then recognition performance should be poor for faces encoded under low-level construal conditions, and for participants who carry out the recognition test following a low-level construal induction. Finally, it served the purpose of providing a replication of both Experiments 1 and 2 with a new sample of participants.

### Method

#### Participants

Eighty-four participants (57 female, *M*_*age*_ = 32.8, *SD* = 15.70) took part in the experiment in exchange for payment of £4^7^. Sample size was determined using GPower with an estimated effect size (f) of 0.67 (derived from Lewis et al., 2009, Experiment 1, encoding X recognition interaction effect) which indicated a required total sample size of 63 (*n* = 21 per between-participants condition) to achieve power of 0.95. Because the construal manipulation used in this study was arguably a more subtle manipulation than the Navon task used in Lewis et al. (2009), we increased the sample by 1/3 (*n* = 28 per between-participants condition).

#### Design and procedure

Participants were seated in individual cubicles. The procedure was identical to that described in Experiment 2 with the exception that, instead of the filler task used in that experiment, participants completed the construal manipulation described in Experiment 1. Thus, the design of Experiment 3 was 3 × 3 × 2 mixed-participants, where Construal Level at Study and Face Orientation at Test were varied within-participants, and Construal Level at Test was manipulated between-participants. All participants were first exposed to 24 study faces, each preceded by a Construal Level at Study manipulation (as in Experiment 2). They were then exposed to one of the three levels of the Construal Level at Test manipulation (as in Experiment 1) before completing the recognition test. The recognition test itself was identical to that used in Experiment 2, with 48 test faces (including 24 “old” and 24 “new” faces, half of which were presented in an upright orientation and half of which were presented in an inverted orientation).

### Results

Three participants over the age of 70, who showed poor overall performance on the recognition task (*M* = 60% correct vs. 72% for younger participants) were excluded from the analyses^8^.

**Figure 3:**
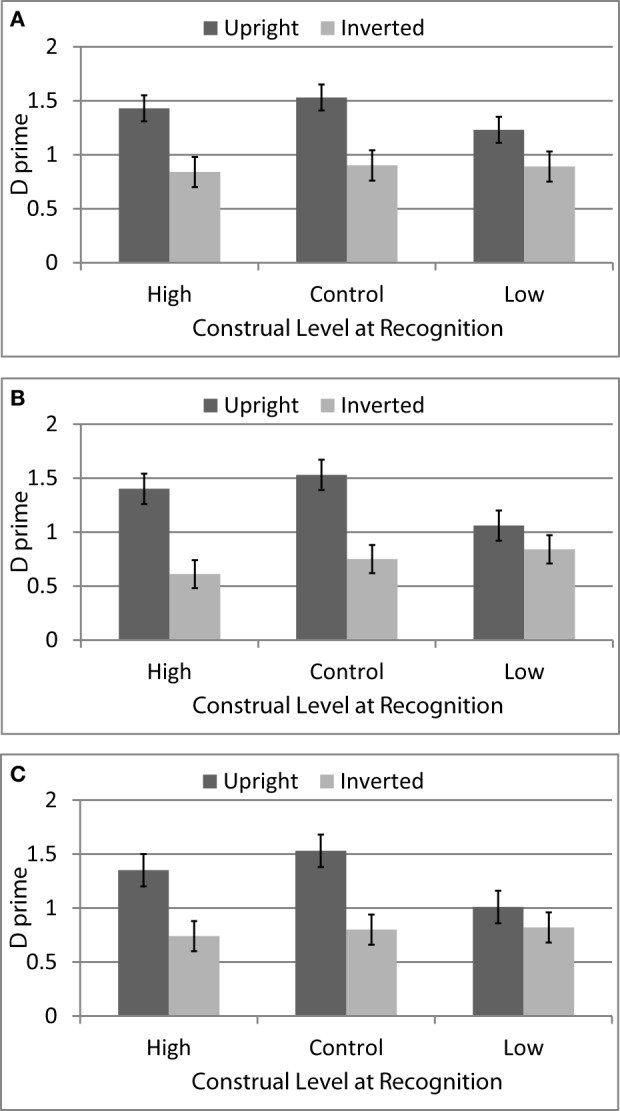
**Face recognition accuracy (average d′ with standard errors) for upright vs. inverted faces following a construal manipulation for faces encoded under high-level construal conditions (A), control conditions (B), and low-level construal conditions (C) (Experiment 3)**.

D′ scores were computed separately for upright and inverted faces in each of the combinations of construal at study and construal at test. Scores were then analyzed using a three-way mixed-model ANOVA where Construal at Study and Face Orientation were repeated measures and Construal at Test was a between-participants factor. Replicating the standard face inversion effect, the analyses yielded a significant main effect of Face Orientation, *F*_(1, 78)_ = 47.21, *p* < 0.001, η_*p*_^2^ = 0.38, *CI:* 0.21, 0.51 (*M*_*upright*_ = 1.34, *SD* = 0.63, *M*_*inverted*_ = 0.80, *SD* = 0.61). As in Experiment 1, this effect was qualified by a significant interaction with Construal at Test, *F*_(2, 78)_ = 3.52, *p* = 0.03, η_*p*_^2^ = 0.08, *CI:* 0, 0.20, as shown in Figure 3. Simple effects analyses indicated that the effect of Face Orientation was significant among participants in the high-level construal at test condition [*F*_(1, 78)_ = 22.70, *p* < 0.001, η_*p*_^2^ = 0.23, *CI*: 0.08, 0.37] and in the control condition [*F*_(1, 78)_ = 27.21, *p* < 0.001, η_*p*_^2^ = 0.26, *CI*: 0.11, 0.40] but not in the low-level construal at test condition [*F*_(1, 78)_ = 3.49, *p* = 0.07, η_*p*_^2^ = 0.04, *CI:* 0, 0.16]. The main effect of Construal at Test was not significant, *F*_(2, 78)_ = 1.05, *p* = 0.36, η_*p*_^2^ = 0.03, *CI:* 0, 0.11.

Replicating Experiment 2, there was also a significant main effect of Construal at Study, *F*_(2, 77)_ = 3.79, *p* = 0.03, η_*p*_^2^ = 0.09, *CI:* 0, 0.21. Pairwise comparisons indicated that faces that were encountered following a high-level construal item were better recognized (*M* = 1.14, *SD* = 0.53) than either those encountered following a low-level construal item (*M* = 1.04, *SD* = 0.58, *p* = 0.03) or those encountered following a control item (*M* = 1.03, *SD* = 0.55, *p* = 03). Recognition rates for faces encountered after low-level construal vs. control items did not differ (*p* = 0.86). As in Experiment 2, Construal at Study did not interact with Face Orientation [*F*_(2, 77)_ < 1, η_*p*_^2^ = 0.01]. Importantly, Construal Level at Study and Construal Level at Test also did not interact [*F*_(4, 156)_ < 1, η_*p*_^2^ = 0.01] nor was there a significant three-way interaction involving Face Orientation [*F*_(4, 156)_ < 1, η_*p*_^2^ = 0.02].

### Discussion

Experiment 3 provided direct replications of the principal findings from Experiments 1 and 2 and clarified the manner in which construal level influences different stages of face learning and recognition. Construal level prior to test influenced both overall level of performance, and the magnitude of the inversion effect, replicating the pattern reported in Experiment 1. In contrast, construal at encoding influenced the recognition of faces regardless of whether they are presented upright or inverted at test, replicating the pattern reported in Experiment 2. Low-level construal appears to particularly detrimental to configural processing when adopted prior to attempting face recognition, as participants in this condition were scarcely better at correctly identifying upright than inverted faces. Collectively, this suggests that while construal level at encoding may influence the quality of a stored representation of a face, construal level when attempting to recognize the face affects the ability to make use of that stored information and of configural information in particular.

The results of Experiment 3 also lend further support to the contention (suggested by Wyer et al., 2012 as well as DeGutis et al., 2013) that the “default” mode of face processing is not necessarily configural for all individuals. In the present experiment, faces encoded under control conditions were not recognized at higher rates than those encoded under low-level construal conditions (both of which were recognized less frequently than those encoded under high-level construal conditions). This would seem to suggest that the “default” level of construal—at least for this sample of participants under these study conditions– was relatively low. The implication of this, of course, is that the default mode of processing faces may, under some conditions, be feature-based rather than configrual.

## General discussion

The three experiments reported here provide consistent evidence that a non-visuo-spatial construal task taken from the social cognition literature affects both the ways in which faces are initially encoded, and the processes employed to later recognize them. Across experiments, high-level construal—whether induced at encoding or at recognition—produced superior face recognition performance relative to low-level construal. These findings have at least four important implications, discussed below in turn.

### How does construal level alter face processing?

The ability to make use of the configural information that is available in a person's face has well-established benefits when it comes to recognizing whether one has seen that person before. Indeed, the research literature provides many examples of experimental manipulations that improve or diminish face recognition performance by altering the likelihood that perceivers are able (or inclined) to utilize configural information (e.g., Yin, 1969; Young et al., 1987; Schooler and Engstler-Schooler, 1990; Macrae and Lewis, 2002; Weston and Perfect, 2005). In the majority of these examples, however, attention to facial features vs. configural facial information is encouraged using visual-spatial tasks that require attention to similar types of information in non-face stimuli (e.g., Navon letters; cf., Schooler, 2002).

In contrast, the present study is the first to provide direct evidence that a manipulation that induces global processing using a completely non-spatial task also results in differences in face processing. Although studies reported by Wyer et al. (2010; see also Hunt and Carroll, 2008) indicated that construal level (as induced by manipulations of temporal distance) altered face recognition performance, they did not specifically identify the processes involved. In the current work, we demonstrated that high-level construal (relative to low-level construal) promotes configural processing, leading to an advantage when it comes to recognizing upright (but not inverted) faces. Thus, like the correlational studies reported by Wyer et al. (2012), non-visual and visual processing styles appear to be linked such that manipulations of one have an impact on the other.

Through what mechanism might construal level alter the way that faces are processed? One likely possibility is that manipulations of construal level induce particular attentional biases that are then generalized to subsequent processing tasks. For example, tasks that induce high-level construal may bias perceivers to attend to global information in general. When they are subsequently asked to recognize previously-seen faces, they may apply this global processing bias to those faces and direct their attention to configural information. Likewise, tasks that induce low-level construal may bias perceivers to attend to detailed information, resulting in a relative neglect of configural information in favor of attending to features in the subsequent face recognition task.

Of course, the fact that the reduced inversion effect produced by participants in our low-level construal condition was driven by impaired performance at recognizing upright faces (rather than improved performance at recognizing inverted faces) is telling. That is, participants in the low-level construal condition did not necessarily attend to facial features to a greater extent—had they done so, one might expect them to be superior at recognizing inverted faces (to the extent that they could retrieve featural information from memory, which would require that they encoded that information during the study phase). Rather, it was their recognition of upright faces that suffered while performance at recognizing inverted faces was similar to that of high-level construal participants. Thus, like individuals who scored high on the AQ scale in Wyer et al.'s (2012) research, it seems that low-level construal participants were unable to make use of configural information but did not necessarily benefit (relative to high-level construal participants) from their presumed attention to featural information. One possibility—alluded to earlier—is that featural information was not encoded (as construal had not been manipulated at that stage) and hence was not available to be used even by those who might be in the best position to take advantage of it.

### What accounts for the high-level construal advantage?

We considered two possible explanations for the superiority of high-level over low-level construal. The first of these draws on conceptually similar work by Lewis et al. (2009) which suggested that a *match* in processing at encoding and at test would promote optimal face recognition. Following this reasoning, we might expect that a match between construal level at encoding and at test would also produce the highest levels of recognition. While the results of our first two experiments do not rule out that possibility, the data produced by Experiment 3 would seem to argue against it. Although inducing high-level construal at test did result in superior recognition of faces encoded under high-level construal conditions, inducing low-level construal at test did not result in an advantage for faces encoded under low-level conditions. In fact, the benefits of having encoded faces under high-level construal conditions were not qualified by the level of construal during the recognition test. At the same time, low-level construal—when induced just prior to test—was associated with substantially weaker inversion effects, which were strongly present under high-level construal conditions. As in previous studies (Wyer et al., 2012; Experiment 1 of the present report), the decrease in the magnitude of the inversion effect was driven entirely by a drop in performance at recognizing upright faces among participants who had been exposed to the low-level construal manipulation prior to the recognition test.

Thus, the evidence from the current set of experiments, taken together, suggests that high-level construal influences face recognition (particularly at the point of deciding whether a test face has been seen before) by promoting global processing. In this report, we have intentionally avoided references to “holistic” processing because of contentions in the literature that the paradigm employed here (i.e., the face inversion effect) is not a direct measure of holistic processing (see Richler et al., 2011b; Susilo et al., 2013). However, recent research from our lab (Wyer et al., accepted) confirms that high-level construal does promote holistic processing; hence the most parsimonious account of the results presented here is that high-level construal produced holistic processing in these studies (and low-level construal undermined it), resulting in differences in the inversion effect.

### What construal level is applied to faces by default?

The studies reported here also provide further evidence in relation to the “default” mode in which faces are processed. While a tacit assumption reflected in much of the face recognition literature is that faces are, by default, processed in a configural manner (cf., Richler et al., 2011b; Wyer et al., 2012; DeGutis et al., 2013), the present experiments present somewhat conflicting evidence. Information about the default level of construal may be best derived from comparing performance under control conditions, where neither high-level nor low-level construal was externally imposed, to performance where one or the other construal level was induced. In Experiment 3, faces encoded under control conditions were later recognized at rates comparable to those encoded under low-level construal conditions. In Experiment 2, recognition rates for control-encoded faces sat roughly mid-way between high-level and low-level encoded faces. Taken together, these studies suggest that “default” construal level when a face is initially encoded is probably variable but more likely to be low-level than to be high-level. When construal level was manipulated prior to the recognition test, the control condition produced recognition rates more similar to the low-level construal condition in Experiment 1, but more similar to the high-level construal condition in Experiment 3. Again, this suggests that the “default” level of construal is likely to vary across participants, such that the proportion of participants operating under either high-level or low-level construal in any given sample of participants is likely to differ.

What does this tell us about the default manner in which faces are processed? At minimum, the results of the present experiments indicate that there are significant individual differences in level of construal, such that overt manipulations are likely to lead to asymmetric changes relative to controls. This suggestion is consistent with the data reported by Wyer et al. (2012; see also Richler et al., 2011a; DeGutis et al., 2013) which identified individual differences in autism-like traits as an important moderator of the face inversion effect (see also Martin and Macrae, 2010). That research demonstrated that configural processing of faces was only spontaneously adopted for participants who did not score highly on the AQ scale. Those who did produce high AQ scores experienced no recognition advantage for upright compared to inverted faces. Likewise, the present set of experiments suggests that, for those individuals who routinely operate at a low construal level, the availability of configural information (in upright faces) will also fail to increase recognition performance.

What these studies cannot tell us, however, is the extent to which these patterns are driven by the nature of the task demands. In all these experiments, participants were exposed to a series of relatively similar images, at a fairly rapid rate, in the knowledge that they would later be tested on their ability to recognize them. In these circumstances, we have shown that the default processing mode is not always configural, and may indeed be quite low level. The extent to which this is a general feature of face-encoding, or represents a strategic response to the demands of the task remains to be further investigated.

### Effects of construal at encoding vs. test

Experiment 3 was successful in replicating the relatively large effect of construal level at test, which interacted with face orientation, and the small effect of construal level at encoding that was independent of face orientation, and showed that these two effects did not interact. This disconfirmed both of our initial expectations. It ruled out our straightforward expectation that construal would have the same effect on processing at encoding and test, and it also ruled out the expectation that performance would be best when construal level at encoding and test matched. Inevitably, this leads us to speculate why this unexpected pattern should have occurred.

We do not have a definitive answer, but two avenues seem worthy of future exploration. One possibility suggested by the differential effects of encoding and test is that construal has a stronger effect on how people retrieve facial details than on how they perceive them. In the recognition test, participants are required not only to process the test stimulus, but also to match this to a stored memory representation. Thus, any effect at test could be due to perceptual processes or retrieval processes. In contrast, construal prior to study involves no retrieval processes, because the to-be-learned stimulus is always present. Thus, construal may have relatively greater impact upon how we reconstruct facial identities rather than how we perceive them. However, whilst this account could explain the pattern seen in Experiments 1–3, it does not offer an intuitive account of related findings (Wyer et al., accepted), in which participants saw the test stimulus approximately 250 ms after the study item.

An alternative account is that construal level has a particular influence on decision processes. At encoding, participants have no decision to make; they merely observe the faces having engaged in high or low-level construal tasks. At test, however, as well as perceiving the test stimulus, and retrieving the study face, they must *decide* whether they match. Even without influencing how people perceive the faces, the construal task may alter the weight assigned to the relative contributions of featural and configural information that arise from the match. High level construal may bias people toward giving more weight to the degree of configural match, whilst low-level construal may bias people toward giving more weight to featural matches. Thus, even without changing perception, construal level could influence face recognition by influencing what aspects of a face the person judges to be most relevant. Notably, this account would apply equally to the effects of construal on the face inversion effect and on the congruency effect from the composite paradigm (Wyer et al., accepted). It is also consistent with other findings in the literature which involve decisions about facial stimuli, including whether a test stimulus is old or new (e.g., Perfect et al., 2008; Weston et al., 2008; Lewis et al., 2009) and whether a test stimulus is the same or different from a study stimulus (e.g., Gao et al., 2011; Curby et al., 2012).

### Limitations and future directions

Before closing, we should note a number of limitations to the current work, which may guide future research in this area. First, we have noted asymmetries in the control conditions across experiments, and have suggested that individual differences in construal level may have contributed to these. Yet we did not measure such individual differences in any of the experiments reported here. Thus, further research is needed to shed further light on the possibility that individual differences (e.g., in construal level or AQ) might account for the asymmetries observed in these experiments.

Although participants responses to the construal manipulations were spot-checked to ensure that they complied with the instructions, we did not include an independent manipulation check to verify that participants were induced to adopt high-level or low-level construal, or that the “how” and “why” manipulations were equally strong. Further research might benefit from such checks.

Finally, further research might clarify the precise mechanism that accounts for the effects of construal level on face recognition. For example, the extent to which construal alters visual attention to global or local features of a face might be examined using eye-tracking during both the study and test phases of the experiment.

## Summary and conclusions

The present work has established a novel link between a manipulation of non-visual processing (i.e., construal level) and the manner in which visual stimuli (i.e., faces) are processed. Across two paradigms, high-level construal promoted configural face processing relative to control or to low-level construal. Importantly, construal appeared to affect processing style only when a recognition or same/different judgment was required, although it also led to overall differences in face memory (not linked to processing style). Further research is needed to specify the specific aspect of judgment (e.g., retrieval or decision) that is altered by construal, as well as the underlying mechanism linking construal level to visual processing style. However, the experiments reported here represent an important step toward determining whether a common mechanism underlies processing of both visual and semantic information.

### Conflict of interest statement

The authors declare that the research was conducted in the absence of any commercial or financial relationships that could be construed as a potential conflict of interest.

## Footnotes

^1^Participants' race was not recorded in any of the experiments reported here; however, they were recruited from a population that is predominantly (over 90%) Caucasian. Any non-Caucasian participants would have been randomly assigned to conditions, and hence own-race effects are unlikely to have systematically influenced any results.

^2^Including this participant in the analyses did not change the outcome.

^3^See Macmillan and Creelman (1991) for detailed information about the use of d′ and c.

^4^Application of Bonferroni corrections renders only a significant difference between high-level and low-level construal.

^5^Participants who had completed Experiment 1 were not eligible for this experiment.

^6^Because the “new” faces in the recognition test were not associated with a specific level of construal, the overall false positive rate was used in computing d' scores for all three conditions. Further, because there was a single false positive rate, it was not possible to examine the effects of construal level on criterion scores (i.e., c) in this experiment.

^7^Participants who had completed either Experiment 1 or Experiment 2 were not eligible for this experiment.

^8^Analyses retaining these three participants yielded very similar results. The direction of mean differences was identical for the two principal results reported below. The Construal at Test X Orientation interaction was significant, *F*(2, 81) = 3.44, *p* < 0.04, η^2^_*p*_ = 0.08. The main effect of Construal at Study was marginally significant, *F*(2, 81) = 2.42, *p* < 0.10, η^2^_*p*_ = 0.06.
